# Metabolic Current Production by an Oral Biofilm Pathogen *Corynebacterium matruchotii*

**DOI:** 10.3390/molecules25143141

**Published:** 2020-07-09

**Authors:** Divya Naradasu, Waheed Miran, Akihiro Okamoto

**Affiliations:** 1 International Center for Materials Nanoarchitectonics, National Institute for Materials Science, 1-1 Namiki, Tsukuba, Ibaraki 305-0044, Japan; NARADASU.Divya@nims.go.jp (D.N.); MIRAN.Waheed@nims.go.jp (W.M.); 2 Center for Sensor and Actuator Material, National Institute for Materials Science, 1-1 Namiki, Tsukuba, Ibaraki 305-0044, Japan; 3 Graduate School of Chemical Sciences and Engineering, Hokkaido University, North 13 West 8, Kita-ku, Sapporo, Hokkaido 060-8628, Japan

**Keywords:** whole-cell electrochemistry, antimicrobial drug assessment, extracellular electron transport

## Abstract

The development of a simple and direct assay for quantifying microbial metabolic activity is important for identifying antibiotic drugs. Current production capabilities of environmental bacteria via the process called extracellular electron transport (EET) from the cell interior to the exterior is well investigated in mineral-reducing bacteria and have been used for various energy and environmental applications. Recently, the capability of human pathogens for producing current has been identified in different human niches, which was suggested to be applicable for drug assessment, because the current production of a few strains correlated with metabolic activity. Herein, we report another strain, a highly abundant pathogen in human oral polymicrobial biofilm, *Corynebacterium matruchotii*, to have the current production capability associated with its metabolic activity. It showed the current production of 50 nA/cm^2^ at OD_600_ of 0.1 with the working electrode poised at +0.4 V vs. a standard hydrogen electrode in a three-electrode system. The addition of antibiotics that suppress the microbial metabolic activity showed a significant current decrease (>90%), establishing that current production reflected the cellular activity in this pathogen. Further, the metabolic fixation of atomically labeled ^13^C (31.68% ± 2.26%) and ^15^N (19.69% ± 1.41%) confirmed by high-resolution mass spectrometry indicated that *C. matruchotii* cells were metabolically active on the electrode surface. The identified electrochemical activity of *C. matruchotii* shows that this can be a simple and effective test for evaluating the impact of antibacterial compounds, and such a method might be applicable to the polymicrobial oral biofilm on electrode surfaces, given four other oral pathogens have already been shown the current production capability.

## 1. Introduction

In the historical perspective, extracellular electron transport (EET) is a respiration strategy by which microorganisms can transfer electrons from the inner membrane, across the periplasm and through the cell-surface to reduce the extracellular electron acceptors such as Fe(III) or Mn(IV) oxides [1,2]. The well-known mechanisms for EET involved in environmental bacteria are via cell-surface *c*-type cytochrome and by redox shuttle molecules, referred to as direct and mediated EET, respectively [3]. Recently, EET-capable bacteria are also identified in the human microbiome *Listeria monocytogenes*, where instead of *c*-type cytochromes, a protein-bound eight-gene locus (including *ndh2*) couples with flavins shuttle electrons to the extracellular electron acceptor [4]. Few other human pathogens with inconclusive or uncharacterized EET pathways such as *Klebsiella pneumoniae*, *Enterococcus avium*, *Enterococcus faecalis*, *Aggregatibacter actinomycetemcomitans* and *Porphyromonas gingivalis* have also been shown to involve in current production/minerals reduction [5,6,7]. Although many pathogens employed fermentation as the source of energy without the requirement of inorganic terminal electron acceptor, EET by pathogens likely facilitate their colonization in the host’s anaerobic environment, which can be of great significance for human health. Therefore, to suggest that the EET system is fairly common in pathogens and involved in their colonization, further investigations are required for pathogens in different human niches.

Environmental microbes capable of EET were initially discovered by researchers more-than 30 years ago [1,2] and have since then envisioned developing large scale cutting-edge applications, including power and energy, water treatment and value-added chemicals, by replacing mineral oxides with electrodes [8,9]. Moreover, at the microscale, current production based biosensors have been developed for environmental pollutants monitoring such as the chemical oxygen demand (COD), heavy metals, etc. [10,11]. However, the current production capability of human pathogens has not been widely employed for potential applications. Given that the pathogens’ current production capability can correlate with their metabolic activity, this can also be used to develop new metabolic assessment tools.

In recent times, bacterial resistance to antibiotics has become a formidable problem for the treatment of many infections and resulted in many clinical deaths and huge economic burdens [12]. Therefore, the discovery of new antimicrobial molecules and the development of easy and effective techniques for their assessment against bacterial colonization is in great demand [13]. Currently, to check the impact of antimicrobial drugs, pathogens’ metabolic activity assay is subjected to microscopic observations, which require gene engineering for expressing the fluorescent protein and complicated interpretations [14]. Among electrochemical analyses for the pathogen’s detection, electrochemical impedance spectroscopy (EIS) has a strong advantage in terms of sensitivity and simplicity, but it does not clearly correlate with metabolic activity and mainly has the benefit of cellular adhesion’s detection on the electrode surface [15]. Thus, the amperometry may be an effective technique for antimicrobial drug selection based on pathogens’ metabolic activity correlation with the current production, and hence the drug’s effectiveness against EET capable pathogens can be assessed. Recent studies showed that *Streptococcus mutans* [16], *Aggregatibacter actinomycetemcomitans*, and *Porphyromonas gingivalis* [6] has the current production ability with the correlation with metabolic activity. These three strains are oral pathogens and the members of the polymicrobial biofilm.

In the present work, we examined the current production capability and its response to the metabolic inhibitors in a highly abundant oral biofilm Gram-positive pathogen *Corynebacterium matruchotii*, which specifically colonize in the supragingival and subgingival plaque. The prevalence of *C. matruchotii* suggests that it plays a highly important role in the plaque community [17]. We conducted a whole-cell electrochemical assay of single-potential amperometry (SA) to study the current producing capability of *C. matruchotii*. We then tested the impact of metabolic inhibitors on the electrochemical activity of *C. matruchotii*. Viability and localization of cells on the electrode surface was examined by scanning electron microscopy and nanoscale secondary ion mass spectrometry (NanoSIMS) [18], respectively. The energy level of potential electron transfer pathways was additionally characterized by differential pulse voltammetry (DPV).

## 2. Results

### 2.1. C. matruchotii’s Metabolism Associated Current Production 

**Figure 1:**
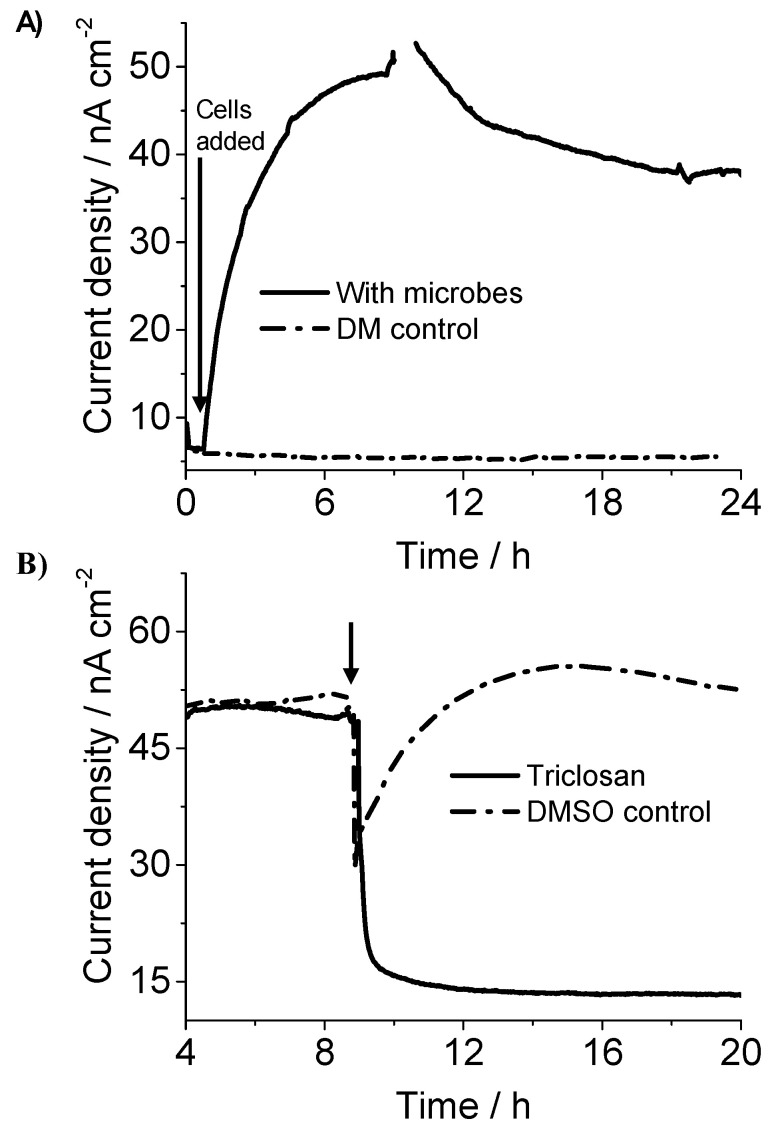
(**A**) Representative current production by *C. matruchotii* in experiments conducted in an anaerobic reactor equipped with indium tin doped oxide (ITO) electrodes (surface area: 3.14 cm^2^) poised at +0.4 V (vs. SHE) in the presence (black solid line) and absence (black dash dot line) of microbes. (**B**) Effect of antibiotic on the current generation. Triclosan, a metabolism inhibitor (black solid line) were added at the points indicated by the arrow to test its impact on *C. matruchotii* electrochemical activity. DMSO control (black dash dot line): DMSO (the same volume as in the triclosan treatment, black line) was added to test the impact of the organic solvent on the current generation.

Electrochemical measurements were performed on *C. matruchotii* to confirm its current production ability by using a three-electrode system by poising the working electrode at +0.4 V vs. the standard hydrogen electrode (SHE). SA measurements showed significant and immediate current production (up to 50 nA/cm^2^) in the presence of 10 mM glucose as the electron donor, after adding *C. matruchotii* cells at 0.1 OD_600_ into the defined medium (DM, electrolyte) (Figure 1A, black solid line). While no current was observed with the electrolyte only, when no bacterial cells were added in the reactor (Figure 1A, black dash dot line), suggesting that *C. matruchotii* cells were most likely involved in the current production. To confirm the association of the current production with cellular metabolic activity, we examined the impact of the metabolic inhibitor antibiotic on the current production of the *C. matruchotii* in the electrochemical system. We used Triclosan, which inhibits glycolysis, i.e., suppresses the metabolism of cells, at lethal concentrations as previously used for oral pathogens [19]. The addition of Triclosan to the *C. matruchotii* electrochemical system during current production after more than 8 h (when current production was stable) resulted in a significant negative impact on current production, i.e., >90% decrease in current production (Figure 1B, black solid line). As Triclosan is insoluble in water, DMSO, an organic solvent, was used for dissolving Triclosan; therefore, the DMSO control experiment was also performed by adding 100 μL (the same volume as for checking the Triclosan impact) in the electrochemical reactor. There was also a sudden decrease in current with DMSO possibly due to the disturbance of colonized cells on the electrode surface; however, the current was increased and recovered within 2 h to the same current pattern as before addition (Figure 1B black dash dot line). In comparison, the current was not recovered even after 10 h for Triclosan added reactor (Figure 1B, black solid line), indicating that metabolism (which was suppressed by Triclosan) is directly linked with current production in *C. matruchotii*. Note that triclosan’s broad spectrum of antibacterial effect on suspension and biofilm cells was already reported that it inhibits the membrane enzymes, glycolysis, and eventually kills the bacteria [20].

**Figure 2:**
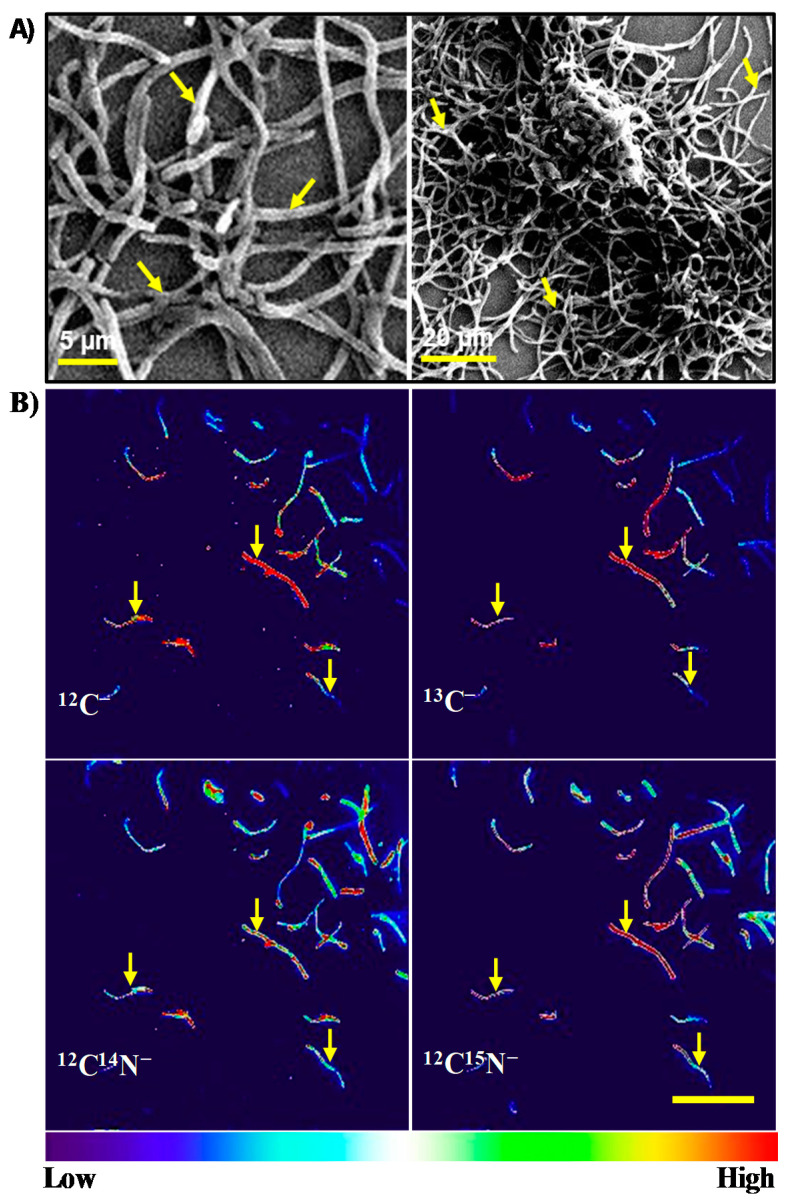
(**A**) Scanning electron micrographs showing *C. matruchotii* cells remained on the ITO electrode after washing processes. (**B**) NanoSIMS images of *C. matruchotii* cells attached to electrodes showing the ^12^C^−^, ^13^C^−^, ^12^C^14^N^−^ and ^12^C^15^N^−^ ion pixel intensities. Scale bar = 20 μm for all four frames. Color gradient bar indicates ion pixel intensity. Arrows indicate the representative cells.

In addition, we confirmed the localization and viability of the cells on the electrode, i.e., at the surface of the ITO electrode (as we placed the ITO electrode at the bottom of the electrochemical reactor) during current production. To this end, we conducted scanning electron microscopy (SEM) for cellular attachment and NanoSIMS for measuring the uptake of ^13^C and ^15^N followed by current production in the presence of ^13^C-glucose and ^15^NH_4_Cl as the labeled carbon and nitrogen source. The attachment of *C. matruchotii* as multi-layered cells was confirmed by SEM on the ITO electrode surface after several washes and dehydration (Figure 2A). After electrochemical experiments, we rigorously washed the electrode surface to remove planktonic, multi-layered or weakly attached cells on the electrode as we need to select the individual cells (regions of interest, ROI) to analyze anabolic ^13^C and ^15^N assimilation of cells by measuring ^13^C/C_total_ (%) and ^15^N/N_total_ (%). NanoSIMS data clearly showed that the electrode attached *C. matruchotii* cells had significant assimilation of ^13^C (31.68% ± 2.26%) and ^15^N (19.69% ± 1.41%; Figure 2B), compared to natural abundance (^13^C: 1.11% and ^15^N: 0.4%), demonstrating that current production was likely associated with the living cell attached at the electrode surface. These results therefore strongly indicate that the current production by *C. matruchotii* reflects cell activity such as metabolism.

### 2.2. Mode of Electron Transfer in C. matruchotii

**Figure 3:**
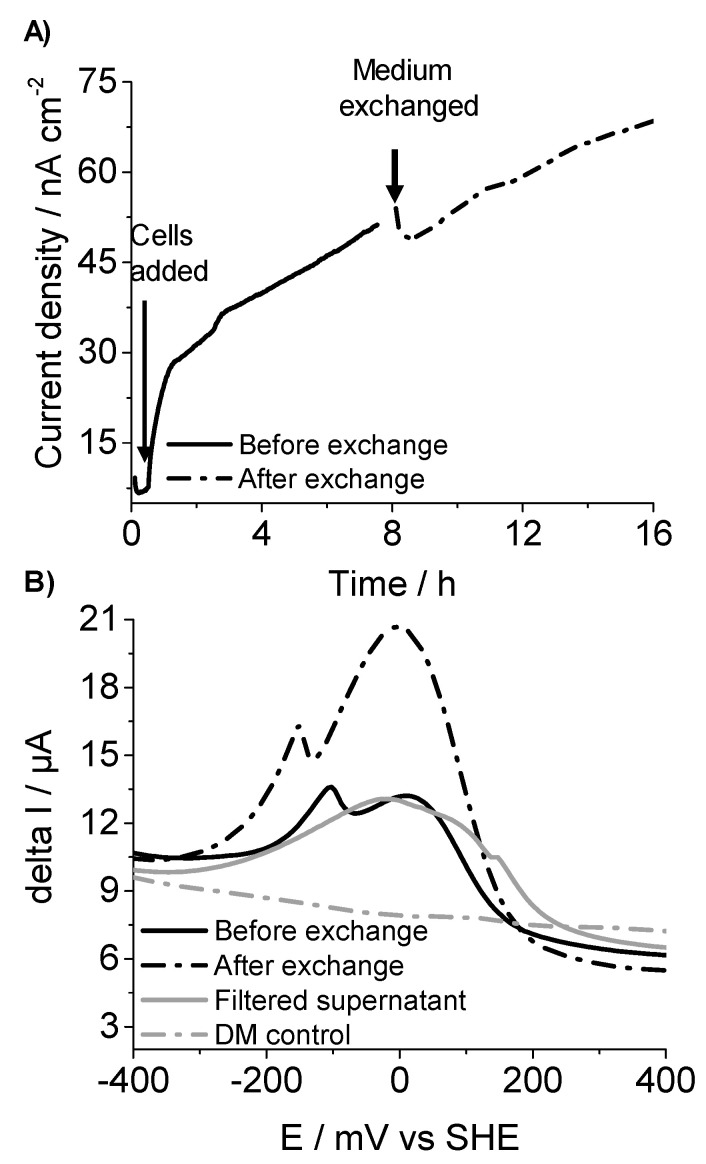
(**A**,**B**) Representative current production versus time and differential pulse voltammetry (DPV) measurements of *C. matruchotii* before (black solid line) and after (black dash dot line) the transfer of spent medium with fresh medium, conducted in an anaerobic reactor equipped with ITO electrodes. DP voltammogram of the filtered supernatant (gray solid line) and defined medium (DM) control (gray dash dot line) was also overlaid for comparison.

To examine the mode of electron transfer that mediate current in *C. matruchotii*, medium swapping experiments were performed [21]. Once a stable oxidation current was observed, the medium from the reactor where *C. matruchotii* cells were attached to electrodes was removed and replaced with fresh DM containing 10 mM glucose as the electron donor. Even after the spent medium replacement with fresh medium and planktonic cells and soluble substrate, which potentially contains redox mediators were removed, current production followed the similar pattern as before the medium replacement (Figure 3A), indicating that the indirect electron transfer mediated by the soluble redox shuttle is not the dominant pathway for the current generation and direct electron transfer was likely the prevailing pathway. This can be attributed to the cell-surface bound redox enzymes or redox shuttles adsorbed to the cell surface. For further confirmation and in-depth analysis of redox molecules, DPV measurements were performed. DPV measurements showed the clear two redox peaks at approximately −125 mV and +25 mV (vs. SHE) with *C. matruchotii* in the reactor (Figure 3B, black solid line). In comparison, no significant peak was observed with electrolyte only (Figure 3B, gray dash dot line), suggesting that *C. matruchotii* contains redox agents that potentially mediate electron transport to the electrode surface. Further, DPV scans from negative to positive potentials showed the increase in both oxidation peak heights (Figure 3B, black dash dot line) after medium exchange compared to before medium exchange consistent with the increase in oxidation current after medium replacement (Figure 3A). The increase in peak heights can be attributed to an increase in cell-surface redox proteins due to culture aging [21]. However, the DPV measurement of a cell-free supernatant of the spent medium also showed a clear broad peak around −20 mV (vs. SHE; Figure 3B, gray solid line). This finding suggested that soluble redox-active compounds were also the components of the observed redox signals. It is important to mention that the previously established modes of EET are somewhat unclear, as observed in this study, intermediate strategies such as adsorption of redox mediators to the cell surface [22] or immobilization of mediators in the biofilm matrix [23] are also possible. Overall, these results suggested that *C. matruchotii* might be a potential EET capable pathogen; however, with an unidentified electron transfer pathway. 

## 3. Discussion

We employed electrochemical measurements to elucidate the current production capability of *C. matruchotii*, a highly abundant pathogen of the supragingival and subgingival oral plaque. SA analysis along with current inhibition by a metabolic inhibitor and single-cell anabolic activity confirmed that the oxidation current was derived from the metabolic activity of *C. matruchotii* cells (Figure 1). However, the current density observed by *C. matruchotii* (50 nA/cm^2^) was lower than EET capable environmental bacteria such as *Geobacter* and *Shewanella* spp. [24,25]. *C. matruchotii* is a Gram-positive bacterium, Gram-positive bacteria usually possesses a thick electron non-conductive cell wall and are known to show weak EET capability in many cases [26,27]. However, as the accumulation of reductive energy suppresses fermentation metabolism in pathogens [28], EET may be an important strategy for them to gain energy for enhancing their colonization and survival by controlling cellular homeostasis.

Although detailed mechanistic studies are important to understand the role of EET in pathogenesis; nevertheless, our results showed that SA could be a unique assay applicable to any EET capable pathogens for qualifying their cellular metabolic activity, and physiological response to antibiofilm compounds. In comparison to conventional techniques involving the microscopic observation of cells, complex sensor systems, and longer cultivation time assays [29,30], our SA strategy can be simple and effective. Moreover, *C. matruchotii* is known for the initiation of oral microbial consortia leading to biofilm formation ranges in size from a few tens to a few hundreds of microns in radius [31,32] and also we observed a multilayered biofilm on the electrode surface in our study by SEM (Figure 2A), while researchers have attributed the decreased antibiotic susceptibility to the reduced antibiotic penetration in biofilms, thus new antimicrobial drugs are in demand, which can enhance their penetration and reach to the bottom of colonized cells [33]. In this regard, our electrochemical platform could also be very effective, because electroactive pathogens may favor growth at the biofilm-electrode interface, and most active cells may be found at the electrode surface [34]; thus, the efficiency of drug’s penetration can be analyzed with electrochemical activity.

It has been well investigated that EET also facilitates long-range electron transport in different natural environments [35,36,37]. For instance, *Ardenticatena maritima* 110S from an iron-rich coastal hydrothermal field has been proposed to couple spatially segregated redox potentials from an electron donor to terminal electron acceptors for current production involving bundled filamentous structure. The multicellular filaments with hundreds of cells arranged end-to-end resulted in the enhanced EET ability to electrodes [36]. Similarly, given that filamentous *C. matruchotii* is discovered as an electroactive oral pathogen in this study and some other oral biofilm pathogens such as *Streptococcus mutans* [16], *Aggregatibacter actinomycetemcomitans* [6], *Porphyromonas gingivalis* [6] and *Capnocytophaga ochracea* [38] have already been shown to have EET capability are well connected with *C. matruchotii*, which nucleates the plaque-characteristic consortium according to the spatial organization in oral biogeography [17], it can be anticipated that long range electron transport is supported by *C. matruchotii* and the whole oral biofilm is electrically conductive. Based on the previous findings that in the oral polymicrobial biofilm, consortium consists of radially arranged taxa, organized around cells of filamentous Corynebacteria with anaerobic taxa in the interior and facultative or obligate aerobes tend at the periphery of the consortium [17], we can purpose that this arrangement may support electrically coupled organics oxidation and oxygen reduction in oral polymicrobial biofilm as in the cases of long-range EET in natural environments. Electrically conductive bacterial nanowires in biofilms associated with bisphosphonate-related osteonecrosis of the jaw (BRONJ) have also been reported [39]. If proved for oral biofilms, this tendency for electrical conductivity of the whole biofilm can be used for antibiofilm drug testing by applying SA assay for the whole oral biofilm. For confirmation, a deeper investigation of the electrical properties of the whole oral biofilm is needed to resolve the conduction mechanism and explore the potential of oral biofilm conduction as a basis for future EET-inspired bioelectronics for oral biofilms and other human niches’ EET pathogenic biofilms.

In terms of the mode of electron transfer, our results showed the possibility of intermediate strategies for current production by *C. matruchotii* (Figure 3), involving adsorption of redox mediators to the cell surface [20] or immobilization of mediators in the biofilm matrix [21]. It has already been reported for environmental bacteria that X-ray crystallography of multiheme cytochromes from the outer membrane of *Shewanella oneidensis* MR1 revealed that there is an interaction between flavins and the cytochromes addition to intramolecular electron transfer pathway [40]. While only a few modes of EET have been proposed to date, these discoveries may not be complete and could be due to a limited number of electricigens discovered and lacking information about detailed mechanism [41]. In this work, our focus is not to identify the genes involved in EET but in the future, it can be found by using mutants obtained by a gene-deletion technique as no EET genes have been identified in *C. matruchotii* to date and their identification may be important for developing strategies for controlling cells growth in oral niches by suppressing their EET capability. 

## 4. Materials and Methods 

### 4.1. Cell Culture Preparation

*C. matruchotii* culture (ATCC 14266) was obtained from Riken BRC, Tsukuba, Japan and pregrown aerobically using the brain heart infusion (BHI) medium (20 mL) supplemented with 1% yeast extract, in falcon tubes at 37 °C. The cells were allowed to proliferate till the late exponential phase where OD_600_ was reached near to 1.0. The grown culture was centrifuged for 10 min at 7800 rpm and 37 °C. The supernatant in the falcon tube was decanted and remaining collected cell pellets were washed with the defined medium (DM) twice. DM composition (gram per liter) was as follows: NaHCO_3_ 2.5, CaCl_2_·2H_2_O 0.08, NH_4_Cl 1.0, MgCl_2_·6H_2_O 0.2, NaCl 10.0, HEPES 7.2 and yeast extract 0.5.

### 4.2. Whole-Cell Electrochemical Analysis

Whole-cell electrochemical measurements were performed in a single-chamber, three-electrode reactor system as previously described [22,42]. Electrolyte used for experiments was 4.8 mL DM containing 10 mM glucose, which was sparged with N_2_ gas for a minimum of 15 min to eliminate the dissolved O_2_ present in the reactor. The *C. matruchotii* cells harvested after washing and centrifugation were diluted with DM for adjustment of OD_600_ to 2.5. Subsequently 0.2 mL of 2.5 OD_600_ cells were added to the reactor containing 4.8 mL of DM to achieve the final OD_600_ of 0.1 by using 1 mL syringe with needle. Potentiostatic condition of +0.4 V vs. a standard hydrogen electrode (SHE) was set for electrochemical measurements, and the reactor was operated without agitation at 37 °C in a COY anaerobic chamber (Grass Lake, MI, USA). Single-potential amperometry (SA) and differential pulse voltammetry (DPV) measurements were conducted with an automatic polarization system (VMP3, Bio Logic Company, France) as reported earlier [22,42]. The following conditions were used for DPV measurements: pulse increments 5.0 mV, pulse amplitude 50 mV, pulse width 300 ms and a pulse period of 5.0 s. Supernatant exchange experiments and scanning electron microscopy (SEM) observations were performed as explained in our previous study [6].

### 4.3. Antibiotic Treatment

The glycolysis inhibitor antibiotic triclosan solution was made by dissolving 30 mg in 1 mL of 100% dimethyl sulfoxide (DMSO) solvent. One hundred microliters of prepared solution was added to the reactor to achieve the final concentration of 0.6 mg/mL [19]. DMSO being an organic solvent was also tested as a control by adding the same volume, i.e., 100 μL to the electrochemical reactor as was used for the triclosan experiment to assess the impact of organic solvent on metabolic activity and current production.

### 4.4. Sample Preparation for NanoSIMS 

Single-cell metabolic assay was conducted for checking the *C. matruchotii* activity on the ITO electrode. *C. matruchotii* cells incubated on the ITO surface at +0.4 V (vs SHE) in DM containing 10 mM ^13^C-glucose as a carbon source and 18.7 mM ^15^NH_4_Cl as a nitrogen source for 24 h were used for quantifying the assimilation, as they can be employed for assessing the cellular metabolic activity owing to their coupling with the extent of anabolism [18,43]. Before carrying out the NanoSIMS, cells attached to the ITO surface were washed thrice, and subsequently fixed in 2.5% (*v*/*v*) glutaraldehyde. Then dehydration of fixed cells was carried out by using the ethanol gradients (30%, 50%, 70%, 90% and 99.5%) and t-butanol (100%) as reported earlier [44]. Thus, the cells that were strongly attached to the ITO surface were analyzed, while planktonic and loosely bound/non-attached cells were removed during multiple washing processes [45]. CAMECA NanoSIMS 50L system (CAMECA, Gennevilliers, France) was used to analyze the *C. matruchotii* cells sample. Briefly, a Cs+ beam approaches the sample and irradiated the four secondary ions (^12^C^−^, ^13^C^−^, ^12^C^14^N^−^ and ^12^C^15^N^−^) emitted from the sample surface. For the substrate assimilation calculation, a raster size of 125 × 25 µm^2^ images of the four secondary ions were recorded, and the images were analyzed by an open source software Fiji (version 1.0). Using the plugin, Open MIMS Image, all signal regions, including cells (with regions of interest, ROI) were selected from each image. The pixel intensities of the four secondary ion images were quantified for assimilated isotopic ratio analysis. NanoSIMS measurements were performed for two experiments and approximately 70 cells were chosen for the calculations. Average and standard error mean (SEM) were determined based on the number of ROIs. The ratio of ^13^C and ^15^N was calculated as follows:

(1) $${}^{13}C\;\mathrm{assimilation}\;(\%)\;=\frac{{}^{12}C}{\left({}^{12}C\;+\;{}^{13}C\right)}\;\times \;100\%$$

(2) $${}^{15}N\;\mathrm{assimilation}\;(\%)\;=\frac{{}^{12}C{}^{15}N}{\left({}^{12}C{}^{15}N\;+\;{}^{12}C{}^{14}N\right)}\;\times \;100\%$$
